# The Hospital World

**Published:** 1911-02-11

**Authors:** 


### THE HOSPITAL WORLD.

### Personalia.

### PRESENTATION TO MISS STUART, OF LIVERPOOL.

MISS STUART, the Lady Superintendent of the Brownlow Hill Poor Law Infirmary, Liverpool, has resigned after twenty-five years' work. Her fine record, to which attention was drawn in Sir Henry Burdett's Report, published in THE HOSPITAL, of January 1, 1910, has been acknowledged by the presentation of a cheque for £120, and of a gold chain and locket. "I do not like presentations," said Miss Stuart, in reply to the speech with which Dr. Alexander accompanied the presentation, "because I feel I am unworthy. You say it is in recognition of my twenty-five years' work here. These twenty-five years have been the happiest of my life. I have enjoyed training you all, more perhaps than you have enjoyed the training." It is interesting to recall that our Report above-mentioned, remarked on this very point: "We have no doubt that a nurse trained at Brownlow Hill, who completes her training and gets her certificate, must necessarily be most efficient." This is perhaps the best summary of a life of personal service which has received such well-deserved recognition at the hands of Liverpool nurses.

PRESENTATION TO A MEDICAL SUPERINTENDENT.—To mark his seven years' service in their midst, Dr. E. C. Austin, of Broughton Astley, recently received an interesting presentation from the parishioners on his leaving to take up his important duties at the Hope Hospital, Salford, where he was recently appointed medical superintendent. The Rev. W. G. Hodges (rector) presided over a large gathering of the doctor's patients and friends, and remarked that to give Dr. Austin the greatest pleasure they had taken him into their confidence and asked him to choose something he desired, which was a massive oak bookcase with bureau centre, which, the rector hoped, would always remind him of his pleasant associations in that district. Mr. C. Cooke, the treasurer of the movement, was then called upon to present Dr. Austin with an illuminated address in the following words: "Presented to Dr. E. C. Austin by the undersigned residents of Broughton Astley and district on his departure, after seven years' residence as their doctor, being a small token of their appreciation and esteem." Here followed the names of 150 subscribers. Dr. Austin was heartily greeted on rising to thank the donors for their gifts, and to convey to them the expression of his wife's and his own thanks for the courtesy he had always received. The balance of the testimonial fund is to be used for the purchase of a suitable present for Mrs. Austin.

### Elections and Appointments.

DR. DONALD has been reappointed assistant house physician for seven years to the Cumberland Infirmary.

MR. W. THORNE, the Mayor of Tiverton, has been elected chairman of the Tiverton Joint Hospital Board.

MR. JULIUS EPHRAIMSON and Dr. Robert Hamilton have been elected vice-presidents of the Bradford Children's Hospital.

THE name of the auditors recently appointed by the Worcester Infirmary should have been Harry Day & Co., and not as incorrectly stated in our last issue.

MR. ROBERT STANTON WOODS, M.D., M.R.C.P., has been appointed physician-in-charge of the Physico-Therapeutical Department and the Electrical Department of the London Hospital. The appointment also includes the charge of the Tyrnauer Baths.

THE General Committee of the Sunderland Infirmary has been elected as follows: Mr. W. A. Bartram, Mr. J. Dale, Mr. T. A. Horan, Mr. F. Lamb, Mr. J. Lloyd, Mr. H. Reed, Mr. R. C. Thompson, Mr. C. R. Walker, and Dr. Waterston.

THE Rev. R. W. Percy Circuitt, the Rev. W. Sawyer, the Rev. T. L. Harry, the Rev. S. Sim, the Rev. W. G. Pearce, Mr. G. Hoare, Mr. J. W. Hill, Mr. P. P. Alexander, Mr. J. P. Decent, Mr. P. Partridge, Mr. W. B. Jackman, Mr. R. W. Edwards, Mr. W. G. Sanders, Mr. J. N. Williams, Mr. J. P. Swaffin, Mr. W. W. Vickery, Mr. R. Jackman, jun., Mr. F. Pearce, Mr. S. Drew, Mr. W. Warren (Churston), Mr. B. A. F. Astley, Mr. W. H. Carter (Churston), S. Buley, jun., Mr. W. H. Wills, Mr. J. L. Arlidge, Mr. J. R. Owen (hon. secretary), Mr. W. J. Sanders, Mr. J. W. Upham, Mr. W. L. Parsons, Mr. D. M. Waterson, Mr. W. P. Poulton, Mr. G. Tryer, the Mr. E. Owen have been elected to the General Committee of the Brixham General Hospital.

THE House Committee of the Newton Abbot Hospital and Dispensary have been re-elected as follows: Commander Jukes-Hughes (Chairman), Dr. E. Haydon, Dr. R. H. Grimbly, Mrs. W. Vicary, Lady Hext, Mrs. Stoneman, Rev. S. Lyne, Dr. W. G. Scott, Dr. A. T. Nisbet, Dr. H. B. Mapleton, Rev. T. C. Walters, and Mr. F. Bulley; and Mr. B. Giles was elected to fill the vacancy caused by the death of Mr. J. Wright. The General Committee were also reappointed, consisting of Mr. E. Parson (Chairman), the members of the House Committee, and the medical officers. Mr. F. F. Card was elected treasurer in succession to the late Mr. W. J. Watts; and Mr. G. D. Woollcombe has been reappointed hon. secretary for the tenth time, with expressions of warm appreciation of his services.

